# Correlation of serum calprotectin with outcome of acute cerebral infarction

**DOI:** 10.1515/med-2025-1207

**Published:** 2025-07-08

**Authors:** HaiYan Li, KaiMeng Zhao, WenWen Yu

**Affiliations:** Department of Geriatrics, The Second People’s Hospital of Lishui, Lishui, Zhejiang, 323000, China; Department of Intensive Care Unit, Huaian Hospital of Huaian, Huaian, Jiangsu, 223200, China; Department of Neurology, Xinchang County People’s Hospital, No.117, Gushan Middle Road, Nanming Street, Shaoxing, Zhejiang, 312500, China

**Keywords:** calprotectin, acute cerebral infarction, severity, outcome

## Abstract

**Objective:**

This study was to investigate the correlation between serum calprotectin (Cal) and the severity and outcome of patients with acute cerebral infarction (ACI).

**Methods:**

Clinical data and serum Cal data were collected from 160 ACI patients and 60 healthy individuals. ACI patients were grouped according to their prognosis. Clinical data, biochemical indicators, and Cal levels were measured. Correlations between serum Cal and National Institute of Health stroke scale (NIHSS) scores, Glasgow Coma Scale (GCS) scores, and hematocrit (HCT) were analyzed.

**Results:**

In ACI patients, elevated serum Cal levels were observed. These levels were positively correlated with NIHSS scores and inversely correlated with GCS scores and HCT. The receiver operating characteristic curve showed that serum Cal had an area under the curve of 0.770 for predicting poor outcomes, with a diagnostic cutoff of 18.01 mg/L. Serum Cal levels over 18.01 mg/L were independently related to poor prognosis for ACI and were identified as an independent risk factor for adverse outcomes.

**Conclusion:**

Serum Cal shows high expression in patients with ACI. Serum Cal has a good predictive value for the outcome of ACI.

## 1 Introduction

Acute cerebral infarction (ACI), also known as acute ischemic stroke, is a sudden interruption of blood supply to the brain, resulting in necrosis of brain tissue [1]. ACI is common and frequently encountered in clinical practice [2]. High rates of mortality and disability in ACI patients represent a significant public health concern [3]. With continuous advances in imaging, the diagnosis of stroke is now based on clinical presentation and neuroimaging (mainly computed tomography (CT) or magnetic resonance imaging (MRI) of the brain) [4]. In most primary hospitals, the use of imaging in ACI is somewhat limited due to the lack of widespread availability of high-end imaging equipment [5]. Studies of serum markers have been reported to reflect different mechanisms associated with ACI, such as brain natriuretic peptide, matrix metalloproteinase-9, cytosolic fibronectin, S-100B, and neuron-specific enolase [6]. The prognosis for people with ACI is determined by a combination of factors, such as the complex relationship between individual differences, pre-existing health problems, and the timing of medical intervention. In addition to intravenous thrombolysis, the timely identification and implementation of other interventions for this group of patients may alter their unfavorable prognosis [7]. It is therefore important to search for serological markers that reflect the extent of disease or prognosis in patients with ACI.

Calprotectin (Cal) is a heterodimer formed by two cytoplasmic proteins, S100A8 and S100A9, which promote inflammation and atherosclerosis progression [8]. Some researchers have found dynamic changes in serum Cal and its correlation with oxidative stress in ACI patients [9]. In addition, inhibition of S100A9 has been shown to inhibit thrombosis in stroke models [10,11]. However, there is limited research on the impact and outcome of Cal on ACI, and no study has comprehensively investigated the correlation between serum Cal levels and the extent and prognosis of ACI. Therefore, the present study was designed to investigate the correlation between serum Cal and brain injury in ACI patients and its potential as an outcome marker for ACI.

## 2 Materials and methods

### 2.1 Patients

One hundred and sixty patients with ACI from March 2021 to March 2023 were used as study subjects.

Inclusion criteria were (1) ACI was diagnosed by the detection of acute neurological deficits and confirmed by CT or MRI [11], (2) patient had a first episode of illness and presented to our hospital within 24 h of the onset, (3) time to first medical contact was <6 h, (4) National Institutes of Health Stroke Scale (NIHSS) score within 24 h of admission, and (5) patients had complete clinical data.

Exclusion criteria included: (1) major cerebral infarction; (2) recurrent cerebral infarction; (3) coagulopathy or other hematological disease; (4) traumatic brain injury infection in the month prior to admission; (5) recent anticoagulant therapy; (6) acute and chronic infectious diseases; (7) severe cardiac, hepatic, and renal insufficiency; and (8) combined with mental diseases, autoimmune diseases, and malignant tumors.

In addition, 60 cases of healthy people were taken as a control group during the same period of physical examination.

### 2.2 Baseline data collection

(1) General data collection: Basic data of ACI patients were collected, including gender, age, BMI, comorbidities (hypertension, diabetes mellitus, and coronary heart disease), NIHSS score on admission, and the time to the first medical contact. (2) Laboratory data collection: patients were admitted to the hospital and 5 mL of fasting venous blood was collected early the next morning before treatment. A Beckman AU5800 automatic biochemical analyzer was used to detect hematocrit (HCT), total cholesterol (TC), triglycerides (TG), low-density lipoproteins (LDL-C), and high-density lipoproteins (HDL-C). The detection of hypersensitive C-reactive protein (hs-CRP) was the same as for Cal, and the human hs-CRP kit was purchased from Elabscience (Wuhan, China). In the control group, fasting venous blood samples were collected during the physical examination, and the aforementioned indicators were assessed.

### 2.3 Determination of Cal in serum

Fasting venous blood (5 mL) was drawn from each ACI patient pre-treatment (without drug treatment). The blood sample was collected from the control group during physical examination. The blood samples were centrifuged at 3,000 rpm for 10 min, and serum was separated and stored at 4°C for testing on the same day and at −80°C for the rest of the samples. Each sample was tested for serum Cal on the day of sampling using an enzyme-linked immunosorbent assay kit (Elabscience). Absorbance values were read at 450 nm using a fully automated microplate reader (RT600, Rayto, Shenzhen, China).

### 2.4 Assessment of ACI severity

NIHSS [12], Glasgow Coma Scale (GCS) [13], and HCT were utilized to assess the severity of brain injury before hospital admission. An NIHSS score of <5 was defined as mild cerebral infarction, and an NIHSS score of ≥5 was defined as moderate-to-severe cerebral infarction.

### 2.5 Treatment

Once admitted to hospital, patients underwent routine examinations, and diagnosis and treatment of acute ACI were initiated according to the recommended protocols of “China Acute Ischemic Stroke Diagnostic and Treatment Guidelines 2018” [12].

### 2.6 Outcome assessment

Functional prognosis at 90 days after ACI was assessed using the modified Rankin Scale (mRs) [13]. Point 0: no symptoms observed; point 1: no major disability despite symptoms; able to perform all standard tasks and activities; point 2: minor disability, unable to perform all previous activities, but able to manage personal affairs independently; point 3: moderate disability, requiring partial assistance, but able to walk unaided; point 4: moderate critical disability, unable to walk unaided, unable to meet needs unaided; point 5: severe disability, bedridden, incontinent, requiring constant care and attention; point 6: death. Patients were categorized into good prognosis and poor prognosis groups based on mRS, where mRS score ≤2 was categorized as good prognosis group and mRS score >2 was categorized as poor prognosis group.

### 2.7 Statistical analysis

The test level (*α*) was set at 0.05 and the test power (1−*β*) at 0.90 by default for statistical analysis. An effect size of 0.5 was selected and the minimum sample size required for the study was calculated as 149 cases using G-Power software. Taking into account a potential loss of 5% of the sample due to visits and other factors, the total sample size was predicted to be 157 cases, which ultimately resulted in the inclusion of 160 patients with ACI in the present study. Using SPSS 22.0 statistical software, normally distributed measures were expressed as mean ± standard deviation and comparisons between the two groups were made using Student’s *t*-test; measures with skewed distributions were expressed as medians (25th–75th percentiles) and comparisons between the two groups were made using the Mann–Whitney *U* test. Count data were expressed as *n* (%) and compared using the *χ* ^2^ test; Spearman correlation analysis was used to analyze the correlation between serum Cal levels and severity of brain injury in patients. Prognostic accuracy was determined using receiver operating characteristic (ROC) curve analysis, with the threshold serum Cal level selected based on the Youden index. Logistic regression analysis was used to assess the prognostic value of serum Cal. Data were visualized using GraphPad 8. *P* < 0.05 was considered a statistically significant difference.


**Informed consent:** The patients signed an informed consent form.
**Ethical approval:** All procedures performed in this study involving human participants were in accordance with the ethical standards of the institutional and/or national research committee and with the 1964 Helsinki Declaration and its later amendments or comparable ethical standards. All subjects were approved by The Second People’s Hospital of Lishui (No. 201902ZJ10).

## 3 Results

### 3.1 Serum Cal in patients

**Figure 1:**
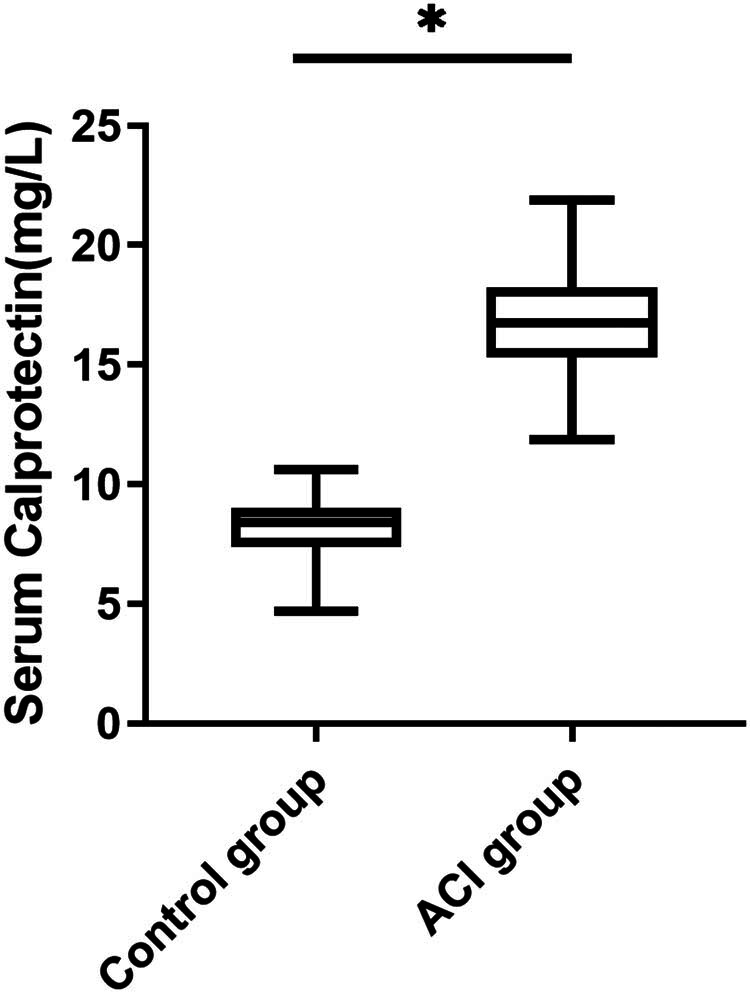
Comparison of serum Cal between ACI group and control group.

There were 93 males and 67 females in the observation group, aged 47–67 (58.68 ± 4.12) years, with a BMI of 20.4–30.7 kg/m^2^ and a mean of 26.54 ± 2.12 kg/m^2^. Among of the control group, 35 cases were male and 25 cases were female, aged 48–65 (58.12 ± 3.34) years old, with a BMI of 18.1–30.9 kg/m^2^ and a mean 25.91 ± 3.04 kg/m^2^. The differences in general information between the two groups were not statistically significant (all *P* > 0.05). Serum Cal (16.79 ± 2.08 mg/L) level was higher in the ACI group than in the control group (8.24 ± 1.16 mg/L) (*P* < 0.05) (Figure 1).

### 3.2 Analysis of serum Cal, clinical data, and biochemical indicators in patients with ACI with different prognoses

Patients were stratified into prognostic groups based on mRS scores: those with scores ≤ 2 (*n* = 112) were classified as having good prognosis, while those with scores > 2 (*n* = 48) were categorized as poor prognosis. The NIHSS score, hs-CRP, and serum Cal levels were significantly higher in the poor prognosis group than in the good prognosis group, while the proportion of patients receiving thrombolytic therapy, GCS score, and HCT was significantly lower than in the good prognosis group (*P* < 0.05). There were no differences in age, gender, medical history, time to first medical contact, proportion of patients receiving routine rehabilitation treatment, proportion of patients taking anticoagulants, or biochemical test results (*P* > 0.05), as shown in Table 1.

**Table 1:** Baseline characteristics of patients with acute cerebral infarction

Indicators	Good prognosis (*n* = 112)	Poor prognosis (*n* = 48)	*P*
Age (mean ± SD)	57.84 ± 5.03	58.36 ± 4.67	0.597
Male (*n*, %)	69 (61.6)	32 (66.67)	0.595
BMI (kg/m^2^)	24.23 ± 1.64	23.78 ± 1.74	0.178
History of hypertension (*n*, %)	63 (56.25)	31 (64.58)	0.383
History of diabetes (*n*, %)	32 (28.57)	12 (25.0)	0.703
History of coronary heart disease (*n*, %)	22 (19.64)	11 (22.92)	0.672
NIHSS score on admission (IQR)	8 (4–13)	12 (6–18)	<0.001
Time to first medical contact (h)	3.52 ± 1.46	3.38 ± 1.31	0.568
GCS score on admission (IQR)	12 (8–15)	10 (8–15)	<0.001
HCT (%)	0.344 ± 0.052	0.2 88 ± 0.042	<0.001
Treatments, *n* (%)			
Thrombolytic therapy	24 (22.32%)	4 (8.3%)	0.043
Routine rehabilitation	91 (81.25%)	38 (79.17%)	0.828
TC (mmol/L)	4.39 ± 1.01	4.55 ± 0.95	0.42
TG (mmol/L)	1.75 ± 0.83	1.54 ± 1.12	0.257
LDL (mmol/L)	2.67 ± 0.91	2.79 ± 0.86	0.502
HDL (mmol/L)	1.19 ± 0.53	1.17 ± 0.32	0.834
UA (g/L)	307.69 ± 94.62	295.56 ± 102.81	0.532
hs-CRP (mg/L)	3.24 ± 0.53	3.58 ± 0.67	0.0036
Cal (mg/L)	12.60 ± 4.01	16.24 ± 5.02	<0.001

Note: Continuous numerical statistical analysis was executed using *t*-test or Mann–Whitney *U*-test, and categorical numerical statistical analysis was executed using *χ* ^2^ test.

Abbreviations: NIHSS, National Institutes of Health Stroke Scale; GCS, Glasgow Coma Scale; HCT, hematocrit; TC, total cholesterol; TG, triglyceride; LDL, low-density lipoprotein cholesterol; HDL, high-density lipoprotein cholesterol; UA, uric acid; hs-CRP, high-sensitivity C-reactive protein; Cal, calprotectin.

### 3.3 Correlation analysis of serum Cal with NIHSS score, GCS scores, and HCT

**Figure 2:**
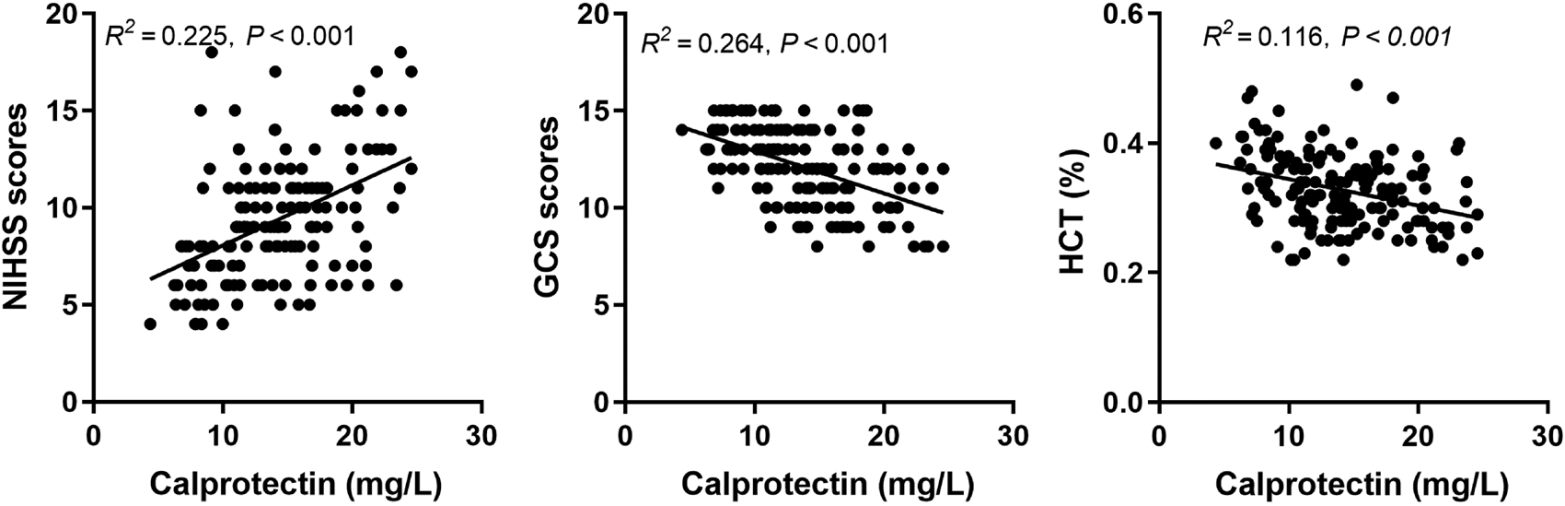
Correlation of Cal levels with NIHSS score, GCS score, and HCT. NIHSS scores, National Institute of Health stroke scale scores; GCS, scores, Glasgow Coma Scale scores; HCT, hematocrit.

This study further analyzed the relationship between serum Cal levels and brain injury severity. The results demonstrated a significant positive correlation between serum Cal levels and NIHSS scores, along with negative correlations with both GCS scores and HCT values, as shown in Figure 2.

### 3.4 ROC curve analysis of serum Cal for predicting poor prognosis

**Figure 3:**
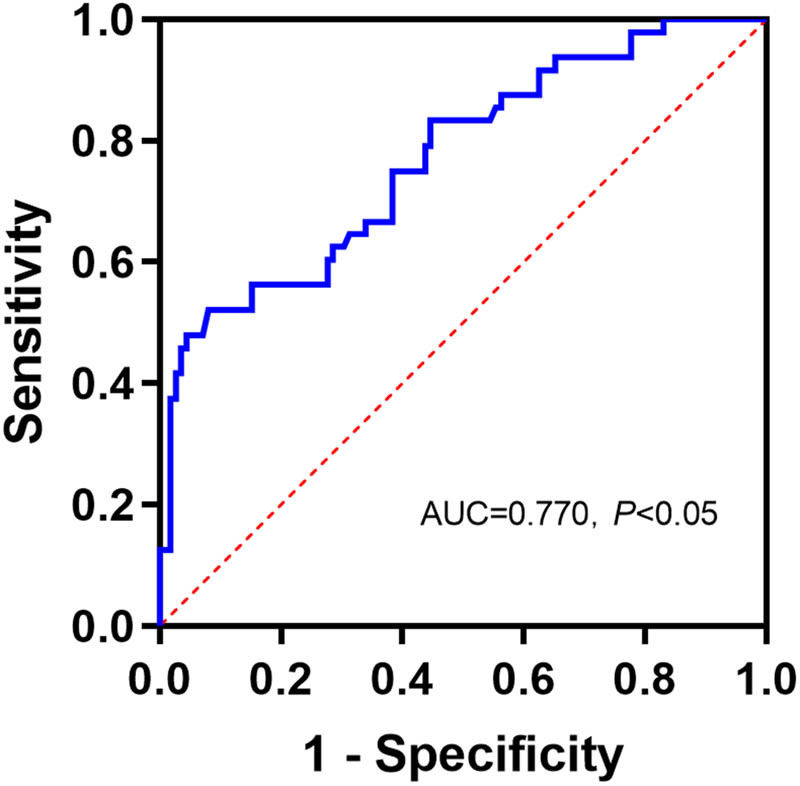
ROC curve of serum Cal for predicting the prognosis of ACI patients. AUC, area under the curve.

According to the ROC curve, the area under the curve (AUC) of serum Cal for predicting poor prognosis was 0.770 (*P* < 0.001), and the cut-off value was 18.01 mg/L, with a sensitivity of 52.08% and a specificity of 91.96%, respectively (Table 2, Figure 3).

**Table 2:** Prognostic value of serum Cal in acute cerebral infarction

Indicator	Cut-off value	AUC	95% CI	*P*-value	False positive rate	False-negative rate
Cal (mg/L)	18.01 mg/L	0.770	0.689–0.852	<0.05	8.04%	47.92%

Cal, calprotectin; AUC, area under curve; 95% CI: 95% confidence interval.

### 3.5 Logistic regression analysis of the prognosis of patients with ACI

Based on the results of the ROC analysis, the above data were finally applied in a multifactorial analysis. The multifactorial logistic regression analysis was performed with age and gender as correction variables, poor prognosis as a dependent variable, and thrombolytic therapy, NIHSS, GCS, HCT, hs-CRP, and serum Cal > 18.01 mg/L as independent variables. Thrombolytic therapy, hs-CRP, and serum Cal > 18.01 mg/L were statistically significant in the model (*P* < 0.05) and were risk factors for poor prognosis of patients, and the administration of thrombolytic therapy was a protective factor against patients’ poor prognosis (Table 3).

**Table 3:** Logistic regression analysis of serum Cal levels on poor prognosis in patients with acute cerebral infarction

Indicators	OR	95% CI	*P*-value
Thrombolytic therapy	0.243	0.076–0.674	0.017
NIHSS	0.819	0.494–1.356	0.437
GCS	0.874	0.613–1.247	0.459
HCT	0.005	0.000–0.012	0.136
hs-CRP	5.346	2.341–15.37	<0.001
Cal > 18.01 mg/L	1.231	1.023–1.486	<0.001

Abbreviations: OR, odds ratio; 95% CI: 95% confidence interval; NIHSS, National Institutes of Health Stroke Scale; GCS, Glasgow Coma Scale; HCT, hematocrit; hs-CRP, high-sensitivity C-reactive protein; Cal, calprotectin.

## 4 Discussion

Given the high rates of disability and mortality associated with ACI, they represent a major threat to human health and quality of life [14]. Particularly in the context of an ageing society, the socio-economic pressures and personal and family burdens of ACI are becoming more pronounced [15]. As a result, the focus of healthcare professionals has expanded beyond traditional pharmacological, interventional, and rehabilitative therapies to include primary and secondary prevention. In addition, professionals are committed to the search for biological markers that can accurately reflect the severity of disease after ACI and help determine prognosis, with the aim of realizing more accurate diagnosis and treatment strategies.

Cal is produced by neutrophils and infiltrating macrophages in the ischemic brain [16]. Some studies have shown an association between Cal levels and several atherosclerosis-related diseases including diabetes mellitus, unstable angina, and coronary heart disease [17–19]. Cal, as one of the diagnostic markers in atherosclerosis combined with chronic periodontitis, is associated with the clinical periodontal index [20]. Cal promotes neuroinflammation and exacerbates the pathology of ACI [21]. Plasma Cal is an independent predictor of 3-month mortality in ACI [8]. These studies suggested a potential association between Cal levels and both the severity and prognosis of ACI. Consistent with prior evidence, elevated serum Cal levels were observed in patients with ACI. In addition, patients with a poor prognosis had higher levels of Cal compared to patients with a good prognosis. S100A9 is associated with adverse outcomes in patients with ischemic stroke (IS) [22]. High plasma Cal concentrations at baseline are independently associated with an increased risk of adverse clinical outcomes at 3 months after IS, and calreticulin may play a role as a prognostic marker for IS [23]. Univariate analysis identified elevated Cal levels as a significant risk factor for poor prognosis.

The NIHSS and GCS are practical and reliable scales that are widely used by clinicians in the early stages of stroke [24]. The NIHSS is commonly used to assess the degree of neurological damage in stroke patients, which is an indicator of neurological deficits and accurately determines the prognosis of patients [24–27]. It has been found that the higher the NIHSS score, the worse the prognosis of patients [28]. In addition, GCS has been used as a means of stratifying brain injury and predicting outcome [29]. In studies of ischemic brain injury, the relationship between HCT and disease severity and prognosis has received attention [30]. High HCT may be associated with increased blood viscosity [31], which may exacerbate ischemia in the brain by making it more difficult for blood to pass through narrowed or blocked blood vessels. Fecal Cal is significantly increased in stroke patients and negatively correlates with GCS [32]. Serum C1q concentrations are positively correlated with infarct volume and NIHSS in patients with ACI [33]. Similarly, in this study, Spearman’s correlation analysis showed that Cal levels were positively correlated with NIHSS scores and negatively correlated with GCS scores and HCT in patients with ACI. The AUC of serum Cal for predicting poor prognosis in ACI patients was 0.770. Furthermore, logistic regression analysis showed that serum Cal > 18.01 mg/dL was an independent risk factor for poor prognosis in patients with ACI. After vascular injury, platelets and neutrophils secrete Cal into the circulation [34]. S100A9 knockdown or neutralization reduces neutrophil recruitment and thrombosis by modulating platelet function without affecting other hemostatic parameters [35]. Previous studies have found that Cal, as an important target in the field of IS therapy, has a mechanism of action that is closely related to the pathophysiological process of brain injury. Specifically, by blocking or inhibiting the activity of Cal, it can effectively reduce the infarct volume in the brain tissue and attenuate the vascular inflammatory response, which in turn promotes the recovery of neurological function and improves the symptoms of neurological deficits [10,36,37]. This study provides further evidence for the potential role of serum Cal in the outcome of ACI. Biochanin A, a blocker of Lrg 1, is involved in attenuating blood-brain barrier damage induced by ischemia-reperfusion injury [38]. Thus, future studies could further investigate whether reducing Cal with specific antagonists would improve ACI.

Understanding the dynamics of release of specific biomarkers is critical to selecting the optimal time point for biomarker measurement. In this experiment, we recorded the serum Cal levels of the patients at the time of admission to hospital, but the dynamic changes in serum Cal levels were not detected. Release kinetics of serum Cal in patients with ACI was demonstrated in a study by Sruk et al. [39]. Whether serum Cal levels would subsequently decrease after regular treatment would need to be investigated by repeating the assay and collecting a large amount of clinical data. In addition, fasting status is difficult to standardize, there is a large individual variation between patients, and the limited time of blood collection may affect the representativeness of the sample and the accuracy of the results. The study was conducted in a single center and therefore the sample size was limited. In addition, there may be subjective influences on the indicators used to categorize the scores, which may affect the scores within the range. Finally, the small number of subjects in the poor prognosis group may lead to imprecise statistical results.

## 5 Conclusion

Serum Cal on admission is positively associated with ACI severity and is an independent risk factor for ACI severity. Serum Cal has good predictive value for the outcome of ACI, and elevated levels of serum Cal are indicative of an unfavorable prognosis in patients with ACI.
